# Below Thermoneutrality, Changes in Activity Do Not Drive Changes in Total Daily Energy Expenditure between Groups of Mice

**DOI:** 10.1016/j.cmet.2012.10.008

**Published:** 2012-11-07

**Authors:** Sam Virtue, Patrick Even, Antonio Vidal-Puig

**Affiliations:** 1 University of Cambridge Metabolic Research Laboratories, Institute of Metabolic Science, Level 4, Box 289, Addenbrooke’s Treatment Centre, Addenbrooke’s Hospital, Hills Road, Cambridge CB2 0QQ, UK; 2 UMR914 Nutrition Physiology and Ingestive Behavior, AgroParisTech, INRA, 75005 Paris, France; 3 Wellcome Trust Sanger Institute, Hinxton, Cambridge CB10 1SA, UK

## Abstract

In this study we investigated the relationship between activity and energy expenditure (EE) in mice. By determining the relationship between activity and EE over a 24 hr period in an individual mouse, activity was calculated to account for 26.6% ± 1.1% of total EE at 30°C. However, when comparing across multiple mice, only 9.53% ± 1.1% of EE from activity appeared to be independent of other components involved in the thermogenic response, suggesting other metabolic processes may mask the contribution of activity to EE. In line with this concept, below thermoneutrality mice still expended a substantial amount of energy on activity; however, at 24°C, 20°C, or 5°C, no independent effect of EE from activity on total daily EE could be detected. Overall these results suggest that when studying mice at temperatures below thermoneutrality, activity is unlikely to explain differences in EE between groups of animals.

## Highlights

► Under standard lab conditions, more-active mice do not expend more energy

## Introduction

The measurement of energy expenditure (EE) is a central feature of studies attempting to investigate the etiology of changes in body weight in rodents. Perhaps the most common method of assessing energy balance is the use of metabolic chambers that can assess variables such as food intake, EE, and activity simultaneously. However, such systems can only give the total EE of a mouse at any given time. Analysis of these data sets raises a fundamental question—how does a change in activity relate to a change in EE? In other words, if a mouse moves twice as much, does it expend 1%, 10%, or 50% more energy?

Answering the question posed above is complicated due to the presence of multiple biological processes that can affect EE in mice. Such processes include basal metabolic rate (BMR) representing metabolic processes that occur at rest at thermoneutrality and in the postabsorptive state; the thermic effect of feeding (TEF), the energy expended on the absorption and processing of nutrients; activity energy expenditure (AEE), the energy that is expended on activity; nonshivering thermogenesis (NST), energy expended predominantly in brown adipose tissue to generate heat to maintain thermal homeostasis; and diet-induced thermogenesis (DIT), energy expended in brown adipose tissue in response to ingestion of food. Importantly, many of these processes occur at the same time (for example, animals must move to eat), making it difficult to discern their specific contribution, since it is only possible to measure the total EE of a mouse. The usual experimental method to assess any of the individual components of energy balance is to measure EE while controlling other variables as comprehensively as possible, as we will now discuss for activity.

Many researchers have made excellent attempts to quantify the contribution that activity *can* make to total daily EE in rodents under conditions when other variables have been controlled for or eliminated. These studies have, in general, relied on some form of regression between very high-resolution measurements of activity and EE. In most of these studies, factors that may confound the contribution of activity to EE were carefully controlled. For example, the majority of studies housed animals at thermoneutrality to minimize NST and were performed in the absence of food or using liquid diets to minimize DIT. These studies have produced a range of estimates for the contribution that activity makes to EE. For rats, three studies have reported 25% (Morrison, 1968), 18% (Brown et al., 1991), and 8% (Girardier et al., 1995). Two studies in mice, conducted at 28°C and 29°C, estimated that the contribution of activity to EE was 38% (Dauncey and Brown, 1987) and 5% (Moruppa, 1990) of daily EE. However, perhaps the more critical question is not if activity *can* contribute to EE under highly controlled circumstances, but if activity actually *does* contribute to differences in EE observed in most metabolic studies conducted below thermoneutrality and under free-feeding conditions.

Most murine metabolic studies are conducted at temperatures between 20°C and 24°C. At 20°C a mouse expends approximately 45% of its daily EE on generating heat. No process for converting chemical energy to mechanical work is 100% efficient, and for a small animal this metabolic efficiency may be as low as 2% (Girardier et al., 1995; Heglund et al., 1982; Taylor and Heglund, 1982), with the majority of wasted energy being lost as heat. It is therefore possible to consider that heat produced from activity may result in a reduction in heat produced from other processes such as NST in brown adipose tissue. Equally, increased thermogenic capacity recruited at ambient temperatures below thermoneutrality (Feldmann et al., 2009; Golozoubova et al., 2006) may result in alterations in the magnitude of other thermogenic processes such as DIT. Finally, increases in EE observed below thermoneutrality are not matched by an increase in activity, suggesting that activity EE becomes a smaller component of the daily energy budget and therefore potentially becomes harder to detect independently of other metabolic processes.

The central aim of this study was to determine if changes in activity were able to drive differences in EE between two groups of animals, for example a group of genetically modified mice and a control group, under “standard laboratory housing conditions” of 20°C–24°C with ad libitum access to food. Overall we found that at temperatures below thermoneutrality, activity did not have an independent effect on total daily EE—in other words, mice that moved more than others did not appear to expend more energy. This is not to say that each mouse did not expend a substantial amount of energy on activity, but that the expenditure induced by movement was masked by changes in other thermogenic processes. The lack of relationship between EE from activity and total EE may have been either due to increased variability in the expenditure of other processes in the total daily energy budget and/or because of a reduction in EE from other metabolic processes that matched EE from activity.

## Results

### The Effect of Total Daily Activity on Daily EE across 30 Wild-Type Mice Housed below Thermoneutrality at 20°C

**Figure 1:**
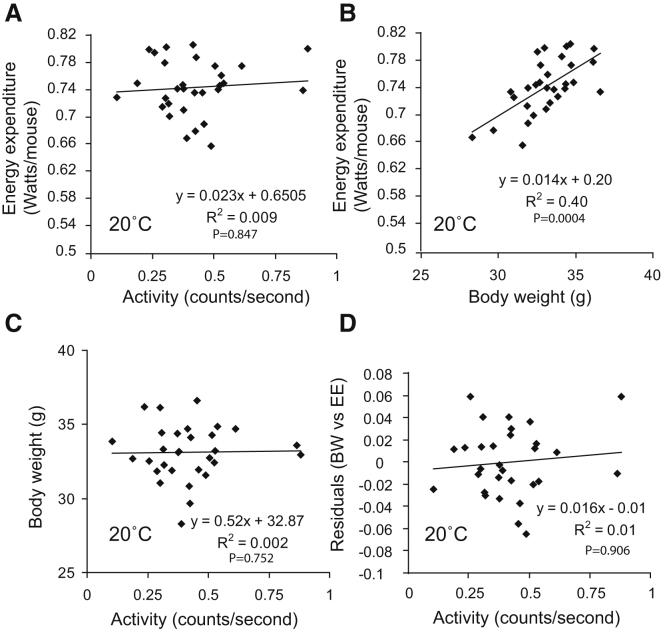
The Impact of Activity on Total Daily Energy Expenditure under “Standard Laboratory Conditions” (A) Activity does not correlate with EE. (B) Body weight correlates with EE. (C) Body weight does not correlate with activity. (D) Correlation of the residuals of EE against body weight with activity reveals no association between activity and EE even when corrected for body weight. All n = 30 mice were housed at 20°C. See also Table S1.

At standard laboratory housing conditions (20°C), activity did not drive gross differences in EE. Figure 1A shows EE and activity data from 30 wild-type (WT) mice measured at 20°C for 69 hr. EE was expressed as joules per second (watts), and average activity was expressed in beam break counts (counts) per second. There was no correlation between activity and EE (Figure 1A). Conversely, Figure 1B presents data from the same animals showing the relationship between body weight and EE. As expected, larger animals expended more energy than smaller animals. Furthermore, body weight did not correlate with activity (Figure 1C). Finally, even when the effect of body weight was controlled for, there was no effect of activity on EE (Figure 1D and see Table S1 online). Overall these data suggested activity had no effect on EE at 20°C.

### The Association between EE and Activity over Time in an Individual Mouse

**Figure 2:**
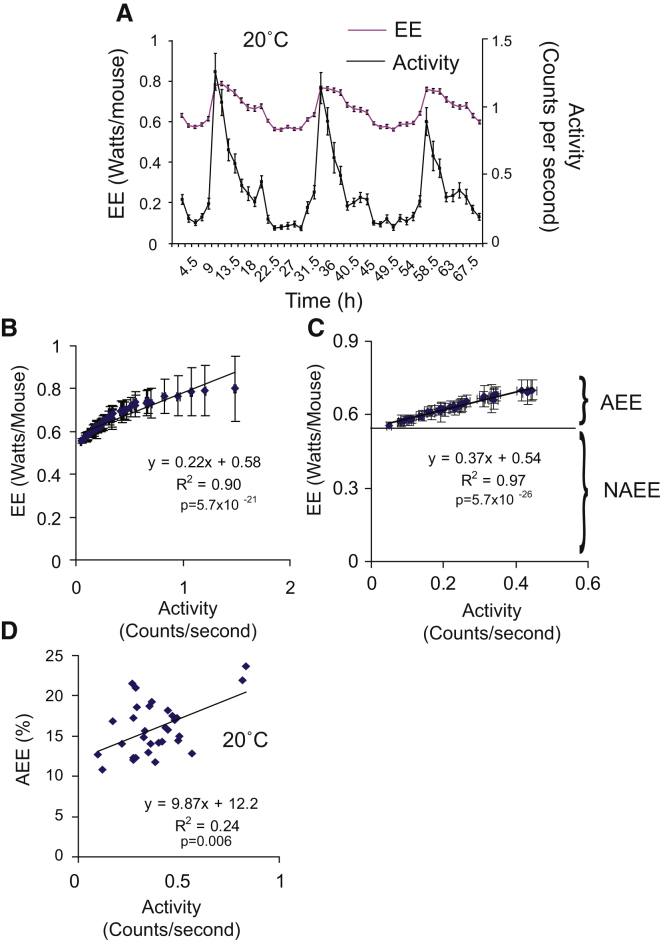
How Circadian Rhythms of Activity Relate to Energy Expenditure (A) Average EE (pink line) and activity (black line) plotted against time over the course of a 69 hr run in 90 min bin intervals. (B) (A) plotted as a correlation between activity and EE in bins. (C) (B) plotted with the maximal five values of activity removed. (D) %AEE plotted against activity. All n = 30 mice were housed at 20°C. Error bars are SEM.

The concept that activity had no effect on EE was apparently at odds with the fact that both EE and activity have circadian patterns in mice. We next sought to investigate the relationship between circadian patterns of activity and EE. EE and activity were binned into 90 min periods for each mouse, and the values for each of the bins were averaged across the 30 mice. Figure 2A shows that when data were analyzed by comparing changes in EE and activity across time, increases in EE were strongly associated with increases in activity. Furthermore, correlating the activity and EE measurements from the 46 bins generated for Figure 1 demonstrated that activity was highly correlated with EE up to 0.45 counts per second (Figure 2B). Regressing activity against EE has been used to determine how much energy is expended as a result of activity in human studies (Ravussin et al., 1986). This technique relies on obtaining the intercept on the y axis (EE) when activity is 0. The difference between EE when activity is 0 and the total EE is assumed to be the energy spent on activity. As the value of EE when activity is 0 is of interest, we considered it valid to remove the highest activity values (those greater than 0.45 counts per second) from Figure 2B. Replotting activity below 0.45 counts per second against EE gave an excellent linear correlation (Figure 2C). We called energy expended below the intercept nonactivity energy expenditure (NAEE), and activity expended above the intercept was termed activity energy expenditure (AEE). Performing this analysis for each of the 30 mice worked well, with an average coefficient of determination of 0.62 ± 0.024, and resulted in an average AEE of 16.2% ± 0.6% of daily EE.

If activity contributed 16.2% of daily EE, then animals which moved half the average amount should expend around 8.1% of their daily EE on activity, and animals moving twice as much as the average should expend 32.4% of their daily EE on activity. This would result in a strong positive correlation between activity and EE, particularly if the effect of body weight was controlled for. However, the results shown in Figures 1A and 1B, which are both derived from the same data as those shown in Figure 2, show there was no correlation between activity and EE in these animals, suggesting that while activity may be contributing to EE, the contribution of activity was not detectable independently of other variables that contribute to EE.

### Calculating the Contribution of Activity to Energy Expenditure that Appears to Be Independent of Other Processes

We next attempted to reconcile the fact that total daily activity did not correlate with EE (Figure 1A), and yet the circadian patterns of activity and EE correlated very well and suggested that activity was contributing 16.2% of daily EE (Figure 2C). Figure 2C relies on regressing activity against EE; however, as the circadian rhythm of EE (Figure 2A) also involves multiple energy-consuming processes including NST, body temperature fluctuations, and DIT, any of these could have been coregulated with activity. To try to determine how much of AEE was actually due to activity, we performed a further analysis. We took the %AEE calculated for each mouse and plotted it against the average activity for each mouse. If %AEE was independent of other metabolic processes, then the regression line for %AEE and activity should pass through the origin, as at zero activity there should have been no EE from activity. However, Figure 2D shows that this was not the case with the majority of AEE (12.2% of 16.2%) not being affected by increasing activity. By subtracting the 12.2% of AEE that was independent of activity from the measured AEE for each mouse, it was possible to calculate the “visible” activity-associated energy expenditure (VAEE). In the case of mice housed at 20°C, VAEE was only 4.0% ± 0.6%. It is worth noting that the correlation in Figure 2D was heavily influenced by two very active mice, without which the VAEE would be 0%.

### Contribution of Activity to EE at Different Temperatures

#### Thermoneutrality

**Figure 3:**
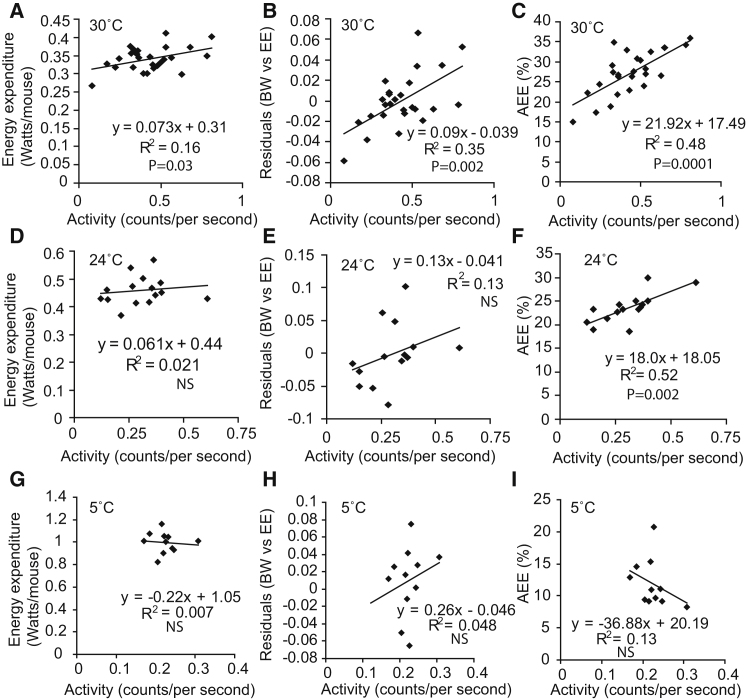
The Effect of Temperature on the Contribution of Activity to Energy Expenditure (A) EE plotted against activity for mice housed at 30°C. (B) Residuals of EE against body weight with activity for mice housed at 30°C. (C) %AEE plotted against activity for mice housed at 30°C. 30°C housed mice, n = 27. (D) EE plotted against activity for mice housed at 24°C. (E) Residuals of EE against body weight with activity for mice housed at 24°C. (F) %AEE plotted against activity for mice housed at 24°C. 24°C housed mice, n = 15. See also Table S1. (G) EE plotted against activity for mice housed at 5°C. (H) Residuals of EE against body weight with activity for mice housed at 5°C. (I) %AEE plotted against activity for mice housed at 5°C. 5°C house mice, n = 11. See also Table S2 and Figure S1.

Logically, if AEE was being masked by alterations in energy dissipated from other thermogenic processes, then measuring animals when NST was switched off (at 30°C) should allow the detection of the contribution of EE from activity. At 30°C there was a weak correlation between activity and EE (Figure 3A). After correcting for body weight, a much stronger correlation between EE and activity was apparent (Figure 3B). Using multiple linear regression (Table S1), an average EE of 0.034 ± 0.0027 W/mouse was attributable to activity or 10.1% ± 0.8% of total EE. In addition to this analysis, we also regressed activity against EE across time in each mouse (using the method shown in Figure 2C). The average coefficient of determination for the mice was 0.75 ± 0.03 and the average AEE per mouse was 26.6% ± 1.1%. Regressing the %AEE for each mouse against activity (Figure 3C) gave a VAEE of 9.53% ± 1.1%, a figure similar to the contribution of activity to EE obtained from regression analysis. This result demonstrated that at thermoneutrality changes in activity could potentially affect total EE between groups of animals.

#### 24°C and 5°C

We next determined AEE at 24°C. We chose 24°C as a temperature that was intermediate between 20°C and 30°C and also represented the upper end of standard laboratory housing conditions. At 24°C there was no detectable correlation between activity and EE (3D) even after correcting for body weight (3E). Conversely, determining AEE (as shown in Figure 2C) for each mouse gave a coefficient of determination of 0.82 ± 0.04 and an average AEE of 23.6% ± 0.8%. Plotting AEE against activity gave a VAEE of 5.5% ± 0.8% of daily EE.

Finally, we looked in conditions of cold exposure. At 5°C there was no detectable contribution of activity to daily EE (Figure 3G) even after correcting for body weight (Figure 3H). On an individual mouse basis, activity was still well correlated with EE with an average coefficient of determination of 0.79 ± 0.064, and AEE for each mouse was 11.9% ± 0.96%. However, AEE was not correlated with activity across the mice studied, suggesting VAEE was 0%.

Of note, when corrected for body weight, the coefficient of variance for the average total EE for each group of mice fell as temperature decreased (Figure S1), suggesting that for beam break data, noise was not obscuring our ability to detect the contribution of activity to EE. Taken together, the results at 30°C, 24°C, 21°C, and 5°C results supported the concept that as temperature fell, the potential for activity to make a detectable impact on total EE became less.

### Alternate Methods for Assessing Activity

#### Running Wheels

**Figure 4:**
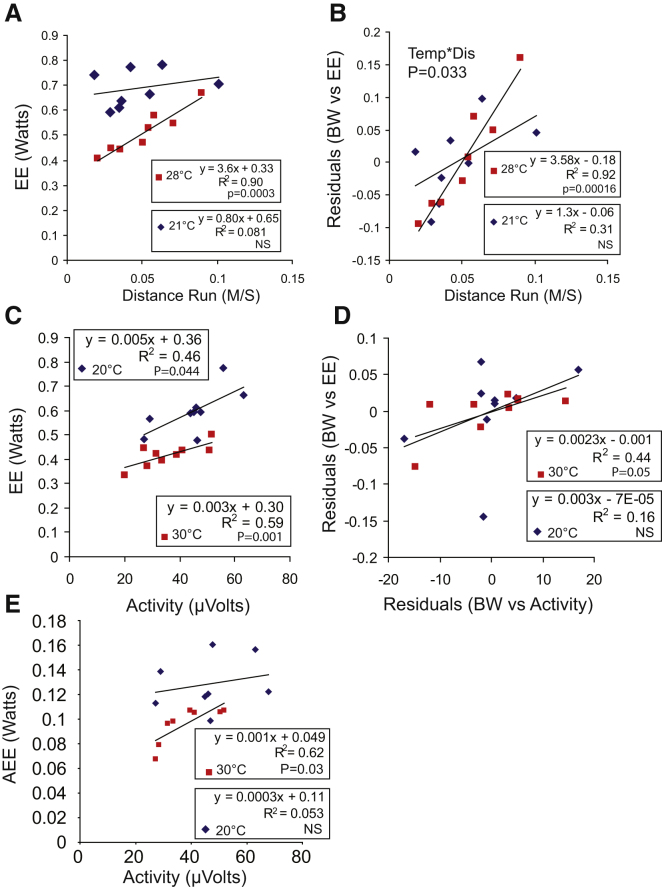
The Relationships between Force Plate and Running Wheel Measures of Activity and Energy Expenditure (A) Distance run plotted against EE. (B) Distance run plotted against residual EE after correcting for body weight. Tem, temperature; Dis, distance run. (C) EE plotted against activity. Activity measured by force plates. (D) Residuals of EE plotted against residuals for activity, both corrected for body weight. Activity measured by force plates 20. (E) AEE plotted against activity (both corrected for body weight; see the Experimental Procedures). Activity measured by force plates.

We attempted to assess activity using two further methods. First, we used running wheels as a method to greatly increase the amount of activity mice were engaging in. As we only possessed 24 hr totals for the distance traveled by mice on the running wheels, we were limited to analyzing the impact of total distance run on EE. We first determined how much a mouse moved in the absence of a running wheel, by assessing ambulatory beam breaks. At 20°C we estimated that the mice traveled a distance of 402 ± 32.8 m/day on average, based on the fact beams were 1.25 cm apart. With running wheels in the cages the mice ran an average of 4.23 ± 0.49 km per day, nearly ten times the level of activity seen in the absence of running wheels. Given the very high rates of activity, we elected to study the mice at lower environmental temperatures to prevent exercise-induced hyperthermia from potentially reducing activity levels. We measured mice at 28°C and 21°C. As expected, correlating activity with EE gave a positive correlation at 28°C but did not give a significant correlation at 21°C (Figure 4A). Correcting for body weight resulted in stronger correlations at both 28°C and 21°C (Figure 4B), though even after this correction the correlation between wheel running and EE did not reach significance at 21°C. Importantly, there was a significant activity^∗^temperature interaction. At 28°C, more than twice as much EE per wheel turn was detectable than at 21°C. Overall this suggested that EE from activity was masked at 21°C by reductions in expenditure from other thermogenic processes, similar to the results observed from beam breaks.

#### Force Plates

To further substantiate our hypothesis, we measured activity using force plates. Beam breaks are limited in two respects with regards to measurement of activity—they are poor at detecting low-intensity activities, and they are also a massless measurement. The amount of energy expended on activity should be proportional to mass, as a heavier object requires more energy to move than a lighter object. To address this issue, we measured activity with force plates while simultaneously recording EE. Activity and EE were measured at a frequency of once every 2 s. Activity correlated with total EE at both 20°C and 30°C (Figure 4C); however, once the effect of body weight on both activity and EE was controlled for, activity only tended toward (p = 0.05) correlating with EE at 30°C (Figure 4D).

As with beam breaks (Figure 2B), there was a strong correlation between activity and EE across time on an individual mouse basis with an average coefficient of determination of 0.81. The average AEE for each mouse was 0.096 ± 0.0064 W at 30°C and 0.14 ± 0.0084 W at 20°C. However, correcting both AEE and activity for body weight and regressing these two variables revealed that AEE was only correlated with EE at 30°C (Figure 4E).

## Discussion

Overall this study demonstrated that at temperatures up to 24°C activity cannot be reliably detected as an independent component of the daily energy budget. This has major implications for interpretation of energy balance in murine metabolic studies, as if activity does not appear as an independent predictor of EE in WT control mice, it is not valid to assume that alterations in activity can drive differences in energy balance between animals with different genotypes. At thermoneutrality, we determined that EE from activity can contribute around 10% to the total daily energy budget. Below thermoneutrality, activity did affect EE when looking at the relationship between activity and EE in an individual mouse across time; however, when comparing EE from activity across multiple mice, the amount a mouse moved did not appear to affect total EE.

### The Confusion Derived from Different Assessments of the Contribution Activity to EE

The widely varying differences in EE attributable to activity that have been reported for rats and mice may in part be due to differences in how EE from activity was calculated. The values of 38% (Dauncey and Brown, 1987) and 5% (Moruppa, 1990) reported for mice were obtained by different methods. The value of 5% was determined by comparing across a group of mice using multiple linear regression. As such, it would only detect EE from activity that appeared independently of other thermogenic processes. Conversely, the value of 38% was obtained by comparing changes in EE that occur in the same mouse across time. Thus the value of 38% should be considered AEE and the value of 5% VAEE. Our own data based on beam breaks show that at 30°C we obtain an average value for AEE of 27% and an average of 10% for VAEE.

### Force Plates

The data from force plate analysis of EE differed slightly from that of beam breaks and running wheels. While an effect of activity on EE (once body weight was controlled for) was still only detectable at 30°C, in this case, the lack of relationship between activity and EE at 20°C appeared to be driven by increases in variability in the activity and EE signals. The reasons for the discrepancy between force plates and beam breaks may be explained by the fact that force plates capture far more data about activity than beam breaks or running wheels. The force plate system we used could detect forces equivalent to moving just 40 mg against gravity. This ability to detect very small forces suggests that low-intensity activities such as grooming and shivering may be detected by force plates, whereas they may not be picked up by beam break systems. In support of this idea, activity from force plates tended to increase as temperature fell, whereas activity based on bream breaks decreased (Table S2), suggesting that low-intensity activities may rise at lower temperatures. The increase in activity at lower temperatures may have in part been a consequence of our study design; it was not possible to fully acclimate animals to 20°C prior to measurement, potentially resulting in shivering which could be recorded by the force plates. Regardless of this, our data suggested that the relationship between activity and EE, even when measuring activity by force plates, was stronger at 30°C than 20°C.

### The Effect of Environmental Temperature on Other Physiological Parameters in Mice

This study adds to a growing body of literature suggesting that murine physiology is strongly influenced by environmental temperature. Several studies exist in which the temperature a mouse model is housed at can dramatically affect phenotypes. For obesity, perhaps the most striking result is *Ucp1* KO mouse, which when compared to WT controls is lean at 20°C but becomes obese at 30°C (Feldmann et al., 2009). In terms of the cardiovascular system, at thermoneutrality, mice, similarly to humans, exhibit significant vagal control of resting heart rate, whereas below thermoneutrality, vagal tone is diminished and resting heart rate is predominantly regulated by the sympathetic nervous system (Swoap et al., 2008). Our results suggest that at thermoneutrality, spontaneous activity in a murine calorimeter accounts for around 10% of daily EE, compared to around 15% for humans in a room calorimeter (Ravussin et al., 1986), whereas at subthermoeneutral conditions voluntary activity was not an independent predictor of daily EE.

### Implications for the Measurement of Activity and Energy Expenditure

It is perhaps worth considering a potential example of how our findings impact on the study of murine energy balance. We can consider a group of knockout (KO) mice and WT controls that have a 30% difference in activity and a 15% difference in EE. Assuming this result was observed when animals were measured at 30°C, then EE from activity (based on our data) would increase from 10% in the WT to 13% in the KO mice, an increase in total daily EE of 3%. The increase of 3% between the groups would be only one-fifth of the total change in daily EE of 15%. Even the above example represents an idealized set of conditions—in most cases, energy balance is assessed below thermoneutrality, where our data suggest the impact of activity on EE would be even less.

Importantly, this study has only been able to cover a very small range of all possible environmental variables and has considered only WT mice. Furthermore, while we have used three separate animal caging systems, there remains a very large array of different sizes and shapes of murine calorimeter. We do not wish to suggest that activity cannot affect total daily EE in all murine calorimetry studies below thermoneutrality. Instead we suggest that before drawing any conclusions about the impact of activity on energy balance, the impact of activity on EE should actually be tested. This can be done rapidly and easily by analyzing body weight (or ideally lean and fat mass), activity, and EE using multiple linear regression. Alternatively, the relationship between activity and EE within individual mice across time can be analyzed to determine activity EE and then the activity EE plotted against the total activity of the mouse.

### Conclusions

In calorimetry systems without running wheels and housed at subthermoneutral temperatures, increases in activity are unlikely to be a factor causing differences in EE when comparing across groups of mice. This is not to say that activity does not contribute to daily EE to a substantial degree, but either that other thermogenic processes such as thermogenesis or DIT are decreased to such an extent that they mask the effect of activity, or that these processes introduce sufficient noise to prevent activity EE from being determined. In order to detect and attribute a contribution of activity to differences in EE between separate mice, we had to house animals at thermoneutrality (30°C). At thermoneutrality, activity was able to account for approximately 10% of daily EE when its contribution was analyzed across a group of animals. Overall, it should not be assumed that changes in activity can explain differences in EE between two groups of mice.

## Experimental Procedures

### Animal Care and Diets

Historical data from the MRC-CORD disease model core were used for this study. Data from 4-month-old male C57Bl/6 mice which had been housed at a density of four animals per cage prior to measurement and housed in a temperature-controlled environment at either 5°C, 20°C, 24°C, or 30°C were used. Animals were kept under a 12 hr light/dark cycle. Food and water were available ad libitum unless noted. All animal protocols used in this study were approved by the UK Home Office and the University of Cambridge. Animals were fed on a normal chow diet (10% of calories derived from fat; D12450B, Research Diets). Animals were acclimated to 5°C, 20°C, or 24°C for at least 3 weeks prior to measurement. Animals measured at thermoneutrality were not acclimated to thermoneutrality but were previously housed at 24°C.

### Indirect Calorimetry and Activity Measurements using Beam Breaks

Animals were placed in a comprehensive laboratory animal monitoring system for measurements at 20°C (Columbus Instruments, Ohio, USA) or Metatrace system for measurements at 5°C, 24°C, and 30°C (Creative Scientific, UK) attached to a custom-built oxygen and carbon dioxide monitoring system (Minimox system built by P. Murgatroyd). Airflow rates were 400 ml/min (20°C, 24°C, and 30°C) or 1,000 ml/min (5°C) measurements of oxygen concentration, and carbon dioxide concentration in room air and air leaving each cage were measured every 18 min. Activity was assessed by beam breaks in both CLAMS and Metatrace systems. Beams in the CLAMS system were 1.25 cm apart. Beams in the Metatrace system were 2.5 cm apart. In both cases, activity measurements were taken to be total beam breaks, rather than consecutive beam breaks.

### Calculation of AEE

For the generation of bins using the CLAMS system, 90 min intervals were used in order to align activity measurements (every 5 min) and EE (every 18 min). For the Metatrace system, 180 min intervals were used in order to align activity measurements (every 10 min) and EE (every 18 min). To determine a cutoff point for linearity, the average of each time bin (i.e., 9 a.m. to 10:30 a.m.) for both activity and EE was taken for all mice studied at a specific temperature. The bin average for EE was regressed against the bin average for activity and a cutoff point determined, above which the relationship between activity and EE ceased to be linear. Subsequently, regression analysis was carried out for each individual mouse between EE and activity. The intercept for EE was calculated and subtracted from the total EE; this value was classed as AEE.

### Running Wheels

Custom-built running wheels with a 33.3 cm internal diameter were manufactured by the Sanger Institute (Hinxton, UK). Mice were individually housed and allowed to acclimatize to the running wheels for 8 days prior to measurement in order to learn to run. Running wheels were placed in Metatrace calorimetry chambers and animals measured for 24 hr. Beam breaks were interrupted by the presence of running wheels, so beam-break activity data could not be simultaneously assessed.

### Force Plate Calorimetry

EE was measured using a custom-built gas analysis system every 2 s (Even and Nadkarni, 2012). Lag induced by the volume of the chamber was corrected for based on the time constant of the chamber. The entire metabolic chamber was mounted on three piezoelectric force meters and activity was taken to be the sum of the three force meters. The activity signal was recorded at 100 Hz, averaged and integrated every 2 s. Animals were housed at 25°C in a 24 hr light cycle. Mice used were C57Bl/6 males, 14 weeks old. Animals were not acclimated prior to measurement of EE. For Figure 4E, AEE was calculated as described above, with activity and EE binned into 15 min intervals. As no correlation between AEE and body weight was detected, to create a body weight-corrected AEE, the intercept and total EE were corrected for body weight and the corrected intercept subtracted from the corrected body weight. Activity was corrected for body weight by adding the residual activity for each mouse (BW versus activity) to the average activity of each group of animals.

### Statistics

Statistics were analyzed using SPSS 17.0 (IBM). Pearson correlations are shown in all figures. For Figure 4B, ANCOVA was carried out to assess activity^∗^temperature interactions using a model containing the interaction term, activity as a covariate, temperature as a fixed factor, and EE as dependent variable. Multiple linear regression analysis was carried out with no selection criteria. All figures show SEM.
