# Supplementary material for: Below Thermoneutrality, Changes in Activity Do Not Drive Changes in Total Daily Energy Expenditure between Groups of Mice
Source: Cell Metab. 2012 Nov 7;16(5):665–71. doi: 10.1016/j.cmet.2012.10.008 (PMC3556741; doi:10.1016/j.cmet.2012.10.008)
Supplement: Document S1. Figure S1, Table S1, and Table S2 [file mmc1.pdf]

Cell Metabolism, Volume *16*

Supplemental Information

Below Thermoneutrality, Changes in Activity Do Not Drive Changes in Total Daily Energy Expenditure between Groups of Mice

Sam Virtue, Patrick Even, and Antonio Vidal-Puig

SUPPLEMENTAL INVENTORY

Figure S1 provides details regarding the absolute energy expenditure corrected for body weight of each of the groups at different temperatures and demonstrates the levels of variance in the energy expenditure at different temperatures. This relates to data from figures 1, 2 and 3.

Table S1 provides regression coefficients for body weight and activity using a linear regression model for animals studied. This provides an alternative statistical method for demonstrating the results shown in figures 1,2 and 3.

Table S2 provides general physical characteristics and raw data values for the groups under study that have been used for all the analyses in the publication.

A

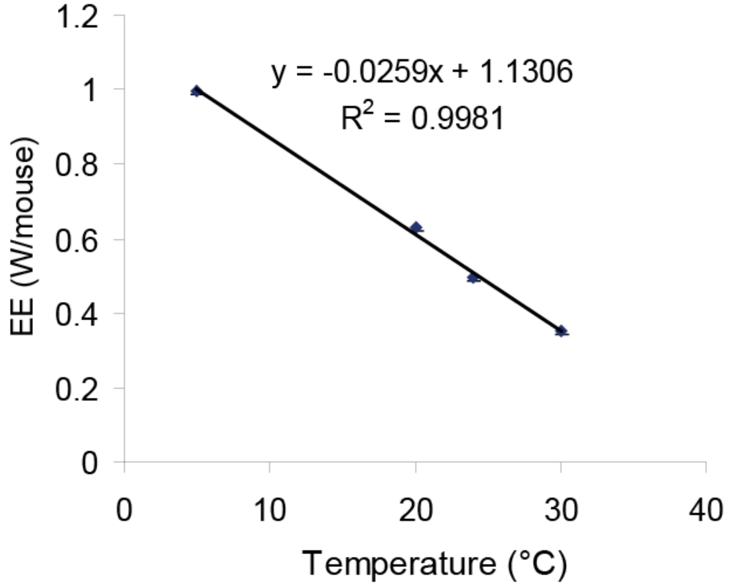

B

Temperature	CV
5°C	3.79%
21°C	6.88%
24°C	8.44%
30°C	10.87%

Supplemental Figure 1. A) Graph showing relationship between EE and bodyweight for mice at different temperatures after correction for body weight using ANCOVA. Mice evaluated at a bodyweight of 30.6g. Data presented +/- SEM. B) Coefficient of Variances for the data shown in A).

Model		Unstandardized Coefficients		Standardized Coefficients	t	Sig.
		B	Std. Error	Beta		
30ºC	(Constant)	-.035	.084		-.421	.677
	Activity	.081	.026	.443	3.095	.005
	BW	.011	.003	.596	4.166	.000
24ºC	(Constant)	.167	.121		1.373	.195
	Activity	.077	.053	.371	1.450	.173
	BW	.009	.004	.597	2.333	.038
20ºC	(Constant)	.195	.106		1.852	.075
	Activity	.016	.036	.065	.444	.661
	BW	.014	.003	.633	4.354	.000
5ºC	(Constant)	.395	.139		2.848	.022
	Activity	.142	.199	.108	.714	.496
	BW	.018	.003	.924	6.084	.000

Supplemental table 1 related to figures 1,2 and 3
Table gives regression coefficients for all groups of animals where activity was measured by beam breaks.
Energy expenditure (Watts/Mouse) is the dependent variable.
Body weight (g) and activity (counts/s) are independent variables.

	CLAMS		Metatrace					
	Average	SEM	Average	SEM	Average	SEM	Average	SEM
Temperature (°C)	20		30		24		5	
Weight (g)	**33.1**	0.33	**29.8**	0.32	**27.6**	0.90	**30.0**	1.33
Activity beam break (counts/s)	**0.37**	0.03	**0.46**	0.03	**0.31**	0.03	**0.22**	0.01
EE (Watts)	**0.66**	0.01	**0.34**	0.01	**0.46**	0.01	**0.99**	0.03
	Forceplate				Running Wheel			
	Average	SEM	Average	SEM	Average	SEM	Average	SEM
Temperature (°C)	30		20		28		21	
Weight (g)	**27.8**	0.95	**27.5**	0.87	**30.2**	0.43	**31.1**	0.77
Activity Force plate (µV/s)	**35.94**	3.55	**44.93**	3.81				
Wheel running (m/s)					**0.051**	0.008	**0.047**	0.009
EE (Watts)	**0.42**	0.02	**0.60**	0.03	**0.51**	0.03	**0.69**	0.03

Supplemental Table 2 relating to figures 1, 2, 3 and 4. Average body weight, activity and energy expenditure for all groups of mice used in this study.
